# Supplementary material for: Comprehensive Annotation and Functional Exploration of MicroRNAs in Lettuce
Source: Front Plant Sci. 2021 Dec 24;12:781836. doi: 10.3389/fpls.2021.781836 (PMC8739914; doi:10.3389/fpls.2021.781836)
Supplement: Supplementary file 2 [file Data_Sheet_8.ZIP › GSM2230747.plot/Lsa-miRN1684_Lsat_1_v5_gn_2_120781.1_701_TPlot.pdf]

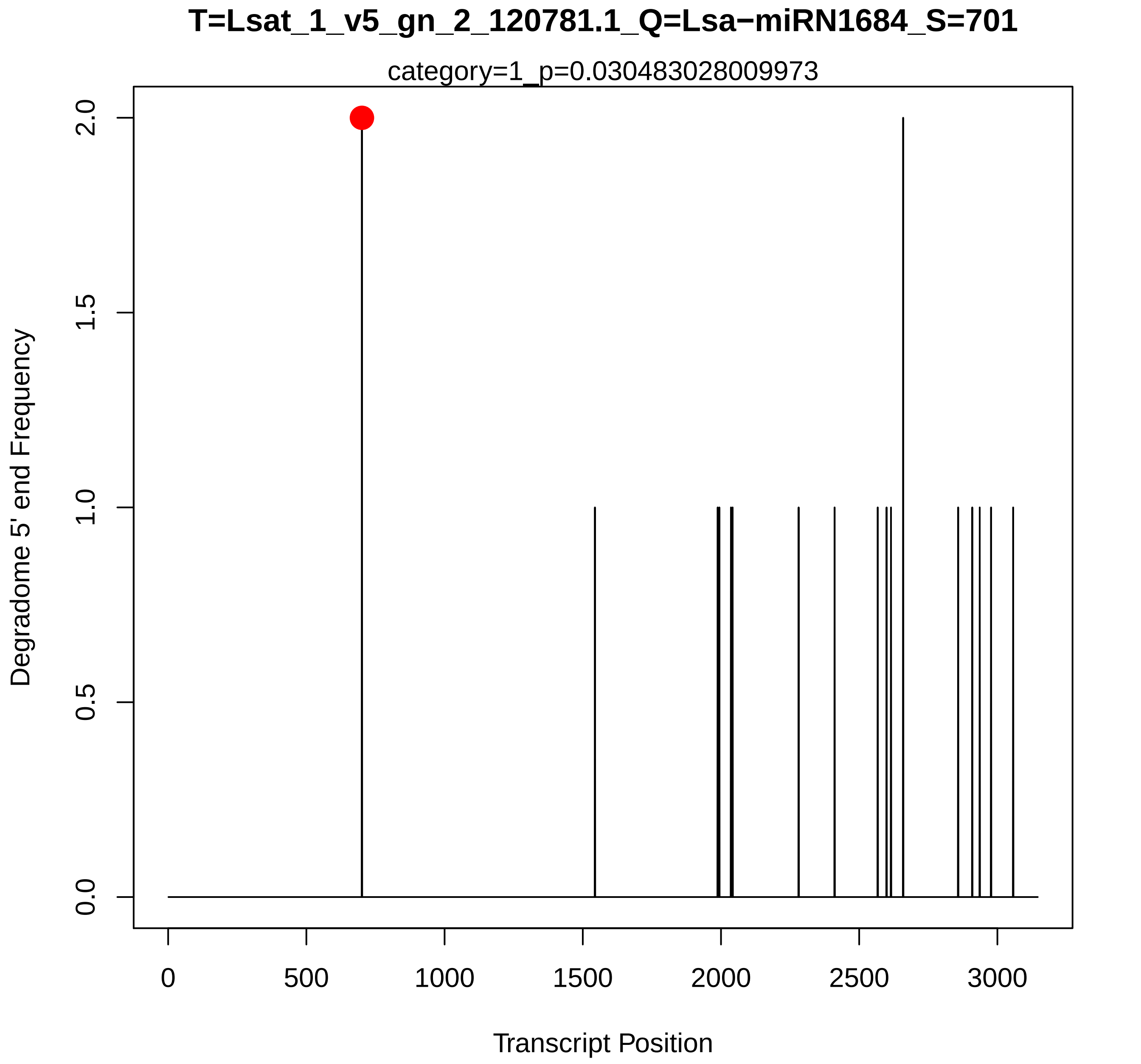

T=Lsat_1_v5_gn_2_120781.1_Q=Lsa−miRN1684_S=701
category=1_p=0.030483028009973
Degradome 5' end Frequency
0.0
0.5
1.0
1.5
2.0
0
500
1000
1500
2000
2500
3000
Transcript Position
